# Cardiac surgery alters the sensitivity of the dynamic interaction between the pituitary and adrenal glands

**DOI:** 10.1186/cc13625

**Published:** 2014-03-17

**Authors:** B Gibbison, J Walker, G Russell, K Stevenson, Y Kershaw, G Asimakopoulos, GD Angelini, SL Lightman

**Affiliations:** 1 University of Bristol, UK; 2 University Hospitals Bristol NHS FT, Bristol, UK

## Introduction

Both ACTH and cortisol are secreted in a diurnal rhythm. Underlying this is an ultradian rhythm of discrete pulses [1] as a result of the feedforward:feedback interactions between cortisol and ACTH [2]. These pulses are critical for normal function; pulsatile and constant infusions yield different transcriptional responses [3] and patients on optimal (nonpulsatile) glucocorticoid replacement have twice the age-related mortality of the general population [4]. We have now characterised the ultradian rhythm and pituitary-adrenal interaction of patients undergoing coronary artery bypass grafting (CABG).

## Methods

Twenty male patients presenting for elective CABG (on-pump and off-pump) were recruited. Blood samples were taken for 24 hours from placement of the first venous access. Cortisol was sampled every 10 minutes, ACTH was sampled every hour and cortisol binding globulin (CBG) was sampled at baseline, at the end of operation and at the end of the 24-hour period.

## Results

**Figure 1:**
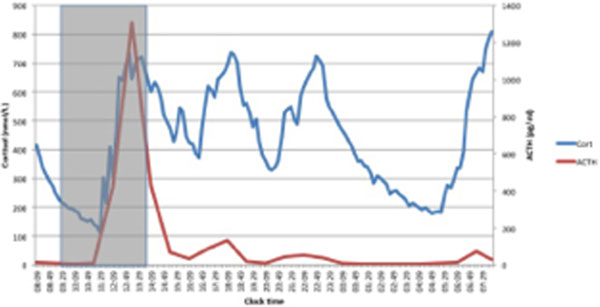


Cortisol and ACTH were pulsatile throughout the perioperative period and the cortisol-ACTH interaction persists (Figure 1). The sensitivity of this interaction (calculated by the ratio of cortisol to ACTH pulse amplitude) changed at about 8 hours post surgery such that the adrenal sensitivity to ACTH increased.

## Conclusion

Both cortisol and ACTH remain pulsatile during and after cardiac surgery and the pituitary-adrenal interaction persists, although the sensitivity of the adrenal glands changes throughout the perioperative period. Our study shows that endogenous glucocorticoid levels reach very high oscillating levels following cardiac surgery, which not only invalidate the interpretation of point measures of adrenal function to diagnose adrenal insufficiency but also demonstrate that constant infusions of hydrocortisone are unphysiological.
